# Spray-Dried Paracetamol/Polyvinylpyrrolidone Amorphous Solid Dispersions: Part I—Stability of Powders and Tablets

**DOI:** 10.3390/pharmaceutics13111938

**Published:** 2021-11-16

**Authors:** Lena Ritters, Yuanyuan Tian, Stephan Reichl

**Affiliations:** 1 Institut für Pharmazeutische Technologie und Biopharmazie, Technische Universität Braunschweig, Mendelssohnstraße 1, D-38106 Braunschweig, Germany; l.ritters@tu-braunschweig.de (L.R.); yuanyuan.tian@tu-braunschweig.de (Y.T.); 2 Zentrum für Pharmaverfahrenstechnik (PVZ), Franz-Liszt-Straße 35a, D-38106 Braunschweig, Germany

**Keywords:** amorphous solid dispersion, tablet, recrystallization, drug release

## Abstract

The formulation of active pharmaceutical ingredients (APIs) in amorphous solid dispersions (ASDs) is a promising approach to improve the bioavailability of poorly soluble compounds. However, problems often arise in the production of tablets from ASDs regarding the compressibility and recrystallization of the API. In the present study, the preparation of spray-dried ASDs of paracetamol (PCM) and four different types of polyvinylpyrrolidone (PVP) and their further processing into tablets were investigated. The influence of PVP type on the glass transition temperature (T_g_) and the physical stability of ASD powders were characterized by differential scanning calorimetry (DSC) and powder X-ray diffraction (XRD). ASD powders with 10 to 30% PCM were stable for at least 48 weeks. PCM contents of 40 to 50% led to recrystallization of the amorphous PCM within a few days or weeks. ASD with PVP/vinyl acetate (VA) copolymer (PVP/VA) was the most unstable and tended to recrystallize in PCM polymorphic form II. This formulation was therefore used for tablet studies. The influence of compression force on recrystallization, crushing strength, and drug release was investigated. Even high compression forces did not affect the stability of the ASD. However, the ASD tablets led to slow release of the API.

## 1. Introduction

The majority of new active pharmaceutical ingredients (APIs) have low water solubility. Amorphous solid dispersions (ASDs) are a common approach to improve solubility and bioavailability [1]. In ASDs, the API is molecularly dispersed in an amorphous carrier. Not only the dissolution properties of the carrier are important for the release of the API but also for the type of API and the drug to polymer ratio. For example, when polyvinylpyrrolidone (PVP) and polyvinylpyrrolidone vinyl acetate (PVP/VA) are associated with low concentrations of API, the drug release is polymer-controlled; formulations with high concentrations of the API tend to result in a release rate similar to that of amorphous API [2]. This property can lead to an improvement in the solubility and dissolution rate. Various synthetic polymers are used as carriers, such as the PVP used here, as well as semisynthetic polymers such as cellulose derivatives [3]. These polymers contribute in different ways to the slow recrystallization of the API. First, synthetic polymers generally have a significantly higher glass transition temperature (T_g_) than the API. Therefore, coprocessing leads to an increase in the T_g_ of the mixture. Second, the polymers impede the molecular mobility of the API particles physically by acting as a protective barrier between them [4]. Third, interactions between an API and polymers can have a stabilizing effect, such as the formation of hydrogen bonds [5].

However, there are currently only approximately 20 commercial amorphous drug products that are available that have been introduced to the market since the 1980s. Although there has been an exponential increase in publications on ASDs [6], they are often limited to powdered products. Nevertheless, the further processing of ASDs into dosage forms is of great interest. In particular, tablets are the most important formulation. It is estimated that currently, 80% of the drugs produced are solid dosage forms such as tablets and capsules [7]. Most of the ASDs on the market are tablets [8]. However, the mechanical stress caused by the compression process can influence API recrystallization. Previous studies have shown that compaction can either increase [9] or decrease [10] the physical stability of ASDs, depending on the drug. In addition, the release of the drug can change due to compaction.

Paracetamol (PCM), the model drug selected for this study, is commonly used as an analgesic and antipyretic. There are three different polymorphic forms of PCM. Recent studies also discuss the existence of a fourth and fifth form of PCM [11]. The commercially available polymorphic form I of PCM is the most thermodynamically stable. Compared to the other two forms, which are orthorhombically arranged, form I has a monoclinic crystal lattice. The PCM molecules of polymorphic form I are arranged in corrugated layers via hydrogen bonds, and as a result of this, they do not have a sliding plane. Therefore, this form is not suitable for direct compression into tablets and tends to cap [12,13]. The structural arrangement in polymorphic form II, on the other hand, is characterized by well-developed slip planes. Weak interplanar interactions connect these planes. This structure leads to plastic deformation, which results in better compactability. However, this form is only metastable. The other forms of PCM are not relevant for tableting, as they are very unstable. Furthermore, the amorphous form of PCM is not stable at ambient temperature due to its low T_g_. Therefore, PCM is an interesting candidate to investigate the influence of ASD formulation on compactability.

The polymers used for the investigations were a short-chain (PVP 12), a medium-chain (PVP 30), and a long-chain (PVP 90) polymer as well as a PVP/vinyl acetate (VA) copolymer (PVP/VA). This polymer has a chain length similar to that of PVP 30 but additionally contains four vinyl acetate moieties, which lowers the T_g_ of the polymer and gives it a lower hygroscopicity.

The objectives of this study were divided into two parts. First, spray-dried ASDs were prepared with PVP 12, PVP 30, PVP 90, and the copolymer PVP/VA. The influence of the different types and concentrations of the polymers on the T_g_ and physical stability of the ASDs with PCM contents of 10 to 60% was evaluated. The most unstable formulation was then used for the second part of the study. In this part, tablets containing ASD or physical mixture (PM) were prepared and analyzed; the effect of different compression forces on the crushing strength, recrystallization tendency, and drug release of ASD and PM tablets with PVP/VA was investigated.

## 2. Materials and Methods

### 2.1. Materials

PCM (polymorphic form I) was a generous gift from STADA (Bad Vilbel, Germany). The polymers PVP/VA (M ~ 45,000 g/mol), PVP 90 (M = 1,000,000–1,500,000 g/mol), and PVP 30 (M = 44,000–54,000 g/mol) were kindly donated by BASF (Ludwigshafen, Germany), while PVP 12 (M = 2000–3000 g/mol) was purchased from Carl Roth (Karlsruhe, Germany). Microcrystalline cellulose 200 (MCC 200) was used as an excipient for the tablet blends and was kindly donated by JRS Pharma (Rosenberg, Germany). Magnesium stearate was purchased from Caelo (Hilden, Germany). Methanol was purchased from Carl Roth (Karlsruhe, Germany), and doubly distilled water was used. Phosphate buffer (pH 5.8) was used for the drug release studies. Potassium dihydrogen phosphate and sodium hydroxide were obtained from Carl Roth (Karlsruhe, Germany).

### 2.2. Preparation of ASDs

The preparation of the ASDs was carried out by spray drying with a Büchi Mini-Spray B-191 dryer (Büchi Labortechnik AG, Flawil, Switzerland) with a two-fluid nozzle (0.7 mm diameter) in co-current airflow mode. PCM and PVP/VA were dissolved in demineralized water at ratios of 1:9 (total concentration of mixture in aqueous solution: 12.0% (*m*/*v*)), 2:8 (6.0%), 3:7 (4.0%), 4:6 (2.4%), 5:5 (1.9%), and 6:4 (1.6%). PCM and PVP 90 and PVP 30 and PVP 12 were dissolved in demineralized water at the ratios of 1:9, 2:8, 3:7 (total concentration of mixtures in aqueous solution: 3.0% (*m*/*v*) each), 4:6 (2.4%), and 5:5 (1.9%). The following conditions were used for spray drying: inlet temperature, 80 °C; outlet temperature, 45 °C to 50 °C; pump rate, 7%; aspirator flow rate, 100%; and airflow, 1100 L/h. The VA50 powder for tablet studies was spray-dried at an inlet temperature of 60 °C. All samples were stored in a desiccator until further use. The nomenclature of the ASD powders can be found in Table 1.

### 2.3. Preparation of PMs

PMs of the drug and polymers (in the same ratios as the ASDs) were prepared in a screw cap glass in a TURBULA^®^ mixer (WAB-GROUP, Muttenz, Switzerland) for ten minutes.

### 2.4. DSC

Freshly prepared and uncompressed powders were subjected to differential scanning calorimetry (DSC) analyzed on a DSC 1 instrument with an FRS5 sensor (Mettler Toledo, OH, USA). Samples of 4–5 mg were weighed in 40 µL aluminum pans with pinholes. Before starting the measurement, the device was equilibrated for 30 min and calibrated using indium. Each sample was analyzed with the following temperature program: In the first step, the samples were heated from 20 °C to 110 °C at a rate of 20 K/min to remove residual water. Then, the temperature was equalized at 110 °C for 5 min before cooling to 20 °C (20 K/min) again. The last step was performed at a heating rate of 10 K/min from 20 °C to 200 °C. All segments were analyzed under a nitrogen atmosphere. DSC thermograms were evaluated with the STARe software (Mettler Toledo, OH, USA).

### 2.5. Storage Conditions

The tablets and the uncompressed powders were stored at 23 °C in a desiccator containing a supersaturated sodium chloride solution to gain 75% RH and at 40 °C and 75% RH in a climate chamber (BINDER GmbH, Tuttlingen, Germany). The tablets were characterized with X-ray diffraction (XRD) after storage for 1, 2, 4, 8, 12, 16, 20, and 24 weeks and, in the case of the uncompressed powder, after 48 weeks. Additionally, the uncompressed powders were stored at 4 °C in a refrigerator without humidity controls.

### 2.6. Powder XRD

XRD patterns were recorded with an X’Pert Pro system (PW3040/60) (PANalytical, Almelo, The Netherlands). The samples were placed in iron spinner cells with a diameter of 12 mm. Tablets were ground in a mortar before analysis. The device had a copper anode and was operated at 40 mA and 40 kV. The samples were analyzed from 3 to 45° 2θ using a scanning speed of 0.023372° 2θ/min and a step size of 0.0167113° 2θ.

### 2.7. Preparation and Characterization of Tablets

#### 2.7.1. Preparation of Tablet Blends and Tableting Process

Tableting studies were performed with PVP/VA-based ASDs. For the preparation of a tablet blend, ASDs were sieved through 0.710 mm mesh. A mixture of 25% ASD or PM and 75% MCC 200 was homogenized in a TURBULA^®^ mixer (WAB-GROUP, Muttenz, Switzerland) for ten minutes. Then, 1% magnesium stearate was added, and the blend was remixed for five minutes. In addition, 100% sieved ASD and PM were directly compressed without any further treatment. Other excipients were not used because their influence on recrystallization behavior should be minimized, and a large number of excipients makes detection by XRD difficult. The compression of powdered mixtures was performed with a Styl’One Evolution compaction simulator (Medelpharm, Beynost, France). Biplane punches 11.28 mm in diameter were manually filled with 200 mg of powder and compacted with compression forces of 5, 10, 20, and 40 kN. Depending on the formulation, dwell times between 0.1 s and 10 s were used. The compositions of tablets are listed in Table 2.

#### 2.7.2. Crushing Strengths

The crushing strengths of the tablets were measured directly after compression using a Dr. Schleuniger Pharmatron model 5Y tablet tester (Dr. Schleuniger Pharmatron Thun, Switzerland).

#### 2.7.3. Drug Release Studies

Drug release studies were performed with freshly compressed tablets with and without MCC 200 on a VK 7000 dissolution system with paddles (VanKel Technology Group, Cary, NC, USA) in 500 mL of phosphate buffer (pH 5.8). Phosphate buffer was manufactured according to Pharm. Eur. 9.0 and was heated to 37 °C and kept at this temperature during the study. The paddle speed was 50 rounds per minute. A sample of 1.5 mL was taken at different times and was replaced with fresh buffer. The samples were analyzed using HPLC. For the evaluation, the t_80%_ value was determined. This value indicated the time after which 80% of the active substance was released and was determined by nonlinear curve fitting of the release curves in OriginLab (Northampton, MA, USA).

#### 2.7.4. SEM

Scanning electron microscopy (SEM) images were taken with a Helios G4 CX (FEI Deutschland GmbH, Dreieich, Germany). The samples were fixed on sample holders on a conductive double-sided adhesive carbon plate. One piece of the surface and one piece of the break edge of each tablet were examined. Before image acquisition, the samples were sputtered with gold using an SCD 030 system (Balzers Union, Balzers, Liechtenstein). Images of different magnifications were taken under high vacuum at 3.00 kV and a beam current of 0.40 nA.

#### 2.7.5. Quantitative Analysis

The amount of dissolved PCM from the release tests was determined by high-performance liquid chromatography (HPLC). The system consisted of a Waters 515 HPLC pump (Waters, Milford, CT, USA), a Midas autosampler (Spark Holland B.V., Emmen, The Netherlands), a column heater, and a Waters 486 UV detector (Waters, Milford, CT, USA). A mixture of 25% methanol and 75% doubly distilled water at a flow rate of 1.5 mL/min was used as the mobile phase. Before use, the solution was degassed in an ultrasonic bath for 30 min. A Hypersil ODS (C18) column (Grom, Ammerbuch-Entringen, Germany) with a particle size of 5 µm and dimensions of 125 mm × 4 mm was used. The column was operated at 23 °C, and a 50 µL sample volume was used. Detection was performed at a wavelength of 243 nm, and PCM was detected at a retention time of 1.2 min. Depending on the expected concentration, the samples were diluted accordingly before measurement. Data analysis was performed with Clarity chromatography software and multipoint calibrations in the range of 0.1 to 55 µg/mL (correlation coefficient >0.999).

## 3. Results

### 3.1. Characterization and Stability of ASDs

**Figure 1:**
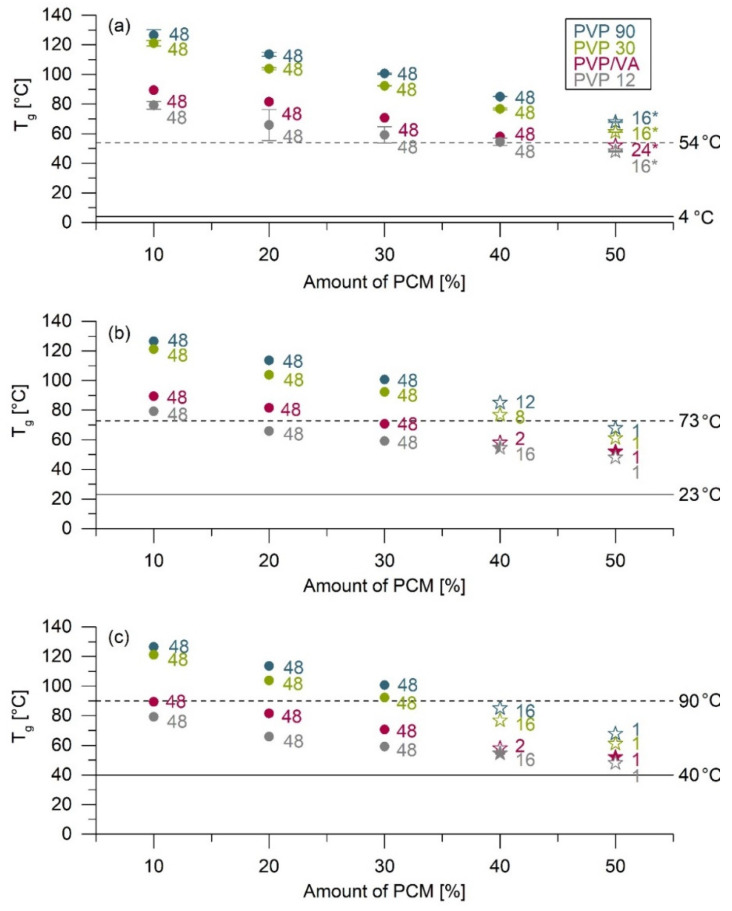
Summary of the glass transition temperature (T_g_) values of ASDs and the stability results obtained by powder X-ray diffraction (XRD) measurement. The stability test was conducted under three different storage conditions: (**a**) storage at 4 °C without humidity control, (**b**) storage at 23 °C/75% RH, and (**c**) storage at 40 °C/75% RH. T_g_: The T_g_ were determined directly after the production of ASDs and are reported as the average ± SD, *n* = 1–2. For clarity, the T_g_ error bars are shown in Figure 1a only. The solid lines indicate the storage temperature, and the dashed lines indicate the temperature 50 °C above the storage temperature. XRD: The XRD results are shown as circles and stars. Circles represent the amorphous state; the numbers indicate the weeks in which the ASDs were still amorphous. Stars represent three types of recrystallization. Open stars show recrystallization in polymorphic form I. Closed stars show recrystallization in polymorphic form II. Semi-closed stars represent recrystallisation in both form I and form II. The numbers indicate the weeks in which recrystallization was detected in the XRD measurements. Numbers with *: initial XRD analysis was carried out 16 or 24 weeks after preparation. Recrystallization may therefore have occurred earlier.

After spray drying, mixtures of PCM and the four different PVP types with 10 to 50% PCM resulted in white powders. The production of an ASD with 60% PCM was not successful because it was very sticky and adhered entirely to the spray tower walls. The T_g_ of the spray-dried products were analyzed by DSC. The recrystallization behavior under different storage conditions was determined by XRD. Figure 1 shows the T_g_ of the ASD powders directly after production. In addition, the times until first recrystallization diffraction peaks were determined with XRD are also presented.

The ASD with PVP 90 showed the highest T_g_ for all PCM concentrations (Figure 1, blue symbols). As the chain length of PVP decreased, so did the T_g_ of the ASD. The copolymer PVP/VA has a chain length similar to that of PVP 30, but the T_g_ of the ASD with PVP/VA was much lower than that of PVP 30 (Figure 1, red symbols).

Directly after production, halos were detected in all powders by XRD. In stability studies, ASDs with 10 to 30% PCM showed no diffraction peaks within 48 weeks under all storage conditions. Additionally, the ASD with 40% PCM stored at 4 °C remained amorphous for at least 48 weeks (Figure 1, circles).

ASD with 50% PCM showed diffraction peaks within one week at 23 °C and 40 °C. The diffraction peaks of ASD VA50 resembled polymorphic form II of PCM (Figure 1b,c, closed red stars). In contrast, diffraction peaks of the other ASD with 50% PCM corresponded to polymorphic form I (Figure 1, open stars). The samples with 50% PCM stored at 4 °C showed diffraction peaks within 16 (PVP 12, PVP 30, PVP 90) or 24 (PVP/VA) weeks of storage (Figure 1a, open stars). However, initial XRD analysis was conducted after 16 or 24 weeks. Recrystallization may, therefore, have occurred earlier.

For ASD with 40% PCM stored at 23 °C and 40 °C, diffraction peaks were observed within 2 to 16 weeks, depending on the polymer and storage conditions (Figure 1b,c). The crystalline pattern of ASD 1240 stored at 23 °C corresponded to both polymorphic form I and form II (Figure 1b, semiclosed gray star). In the case of storage at 40 °C, mainly diffraction peaks from PCM polymorphic form II could be detected (Figure 1c, closed gray star).

Overall, the ASD with PVP/VA showed the fastest recrystallization, while the ASD with PVP 12 tended to remain amorphous for the longest time. However, it should be noted that the stability studies of ASDs with PVP of different chain lengths only showed minor differences.

**Figure 2:**
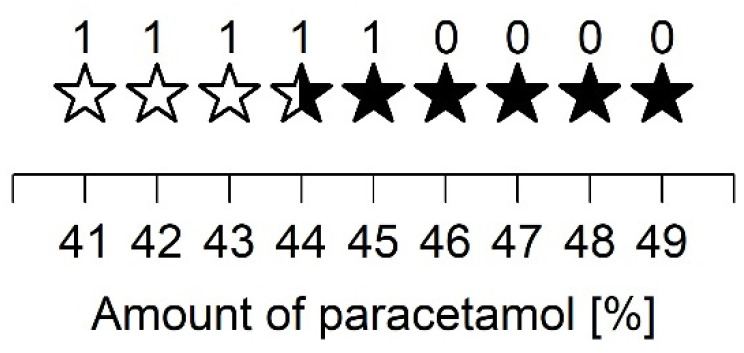
Stability of ASDs prepared with PVP/VA during storage (storage conditions: 23 °C/75% RH and 40 °C/75% RH; no differences were observed between the two storage conditions). Stars represent three types of recrystallization. Open stars show recrystallization in polymorphic form I. Closed stars show recrystallization in polymorphic form II. Semiclosed stars represent recrystallization in both form I and form II. The numbers indicate the weeks in which recrystallization was detected in the XRD measurements.

To investigate the recrystallization behavior of ASDs with PVP/VA in more detail, a further stability study was conducted on ASDs containing 41 to 49% PCM (Figure 2). This investigation found that ASD with PCM quantities of 46 to 49% showed diffraction peaks of polymorphic form II directly after preparation. The ASDs with relatively low PCM amounts were amorphous directly after preparation but showed diffraction peaks within one week of storage. PCM amounts of 41 to 43% led to recrystallisation in polymorphic form I, while ASD VA44 showed diffraction peaks of both form I and form II. After six months of storage, ASDs with 44%, 45%, and 49% PCM showed single diffraction peaks of form I, while no further changes could be observed in the XRD investigations for all other ASDs.

### 3.2. Characterization and Stability of ASD Tablets

#### 3.2.1. Crushing Strength

The most unstable system possible should be used for the tablet studies. Since ASD powders with PVP/VA were the most unstable in the stability studies, these ASDs were used for further investigations. ASD powders with 30% PCM remain amorphous for at least 48 weeks. In contrast, ASDs with 40% and 50% PCM show recrystallization within a short time. Since these ASDs represent the boundary between stable and unstable preparation, they were selected for the tablet studies.

**Figure 3:**
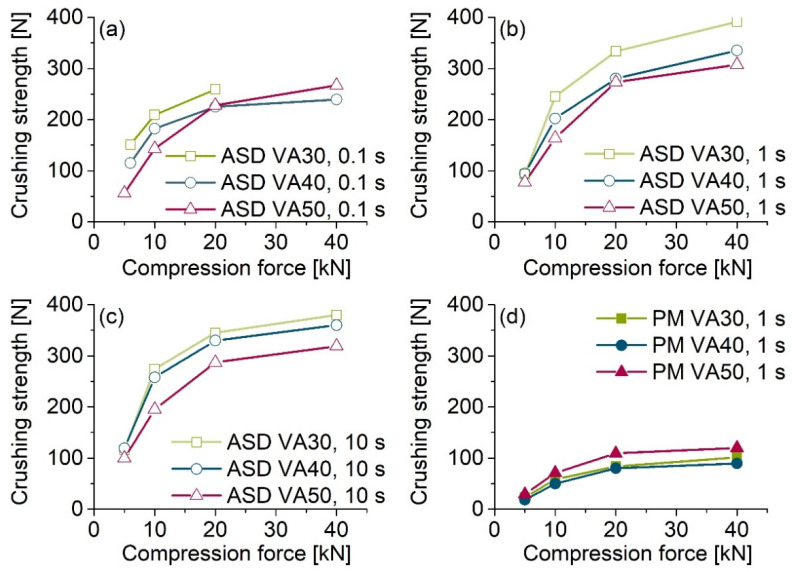
Crushing strengths of ASD and PM tablets with MCC 200 directly after preparation as a function of compression force. The ASD tablets were compressed with three different dwell times: (**a**) 0.1 s, (**b**) 1 s, (**c**) 10 s, and (**d**) PM tablets compressed with a dwell time of 1 s. Due to the small sample quantity, measurements were performed once per sample.

**Figure 4:**
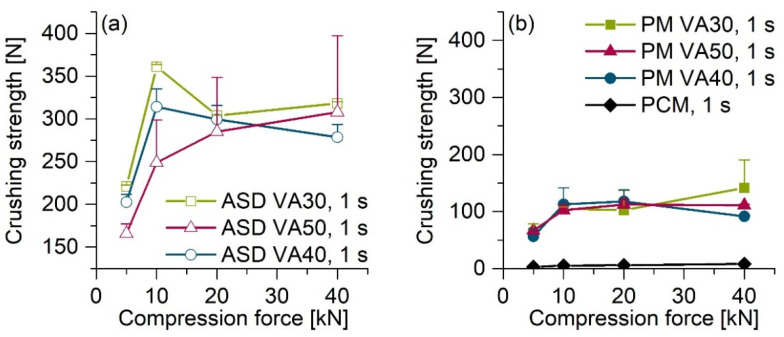
Crushing strengths of pure ASD and PM tablets without MCC 200 directly after preparation as a function of compression force: (**a**) pure ASD tablets compressed with a dwell time of 1 s, (**b**) pure PM and PCM tablets compressed with a dwell time of 1 s; average ± SD, *n* = 2. For clarity, only the upper error bars are displayed.

All of the ASD tablets showed three to four times higher crushing strengths than the corresponding PM tablets. The tablets with MCC 200 (Figure 3) showed lower crushing strengths at lower compression forces than the tablets without MCC 200 (Figure 4). An increase in the compression force led to a continuous increase in the crushing strengths of the tablets with MCC 200. Maximum crushing strengths of approx. 300–350 N for the ASD tablets and approx. 100 N for the PM tablets were achieved for both tablet types.

The ASD tablets compressed at a dwell time of 0.1 s (Figure 3a) had the lowest crushing strengths. Within a formulation, an increase in the dwell time led to an increase in the crushing strengths, whereby the difference between the 1 s dwell time (Figure 3b) and 10 s dwell time (Figure 3c) was relatively small. The crushing strengths of tablets with a relatively high PVP content tended to be slightly higher than those of tablets with a relatively low PVP content. Compression of crystalline PCM resulted in tablets with crushing strengths <10 N for all of the compression forces (Figure 4b, black line).

#### 3.2.2. Recrystallization of Tablets

**Figure 5:**
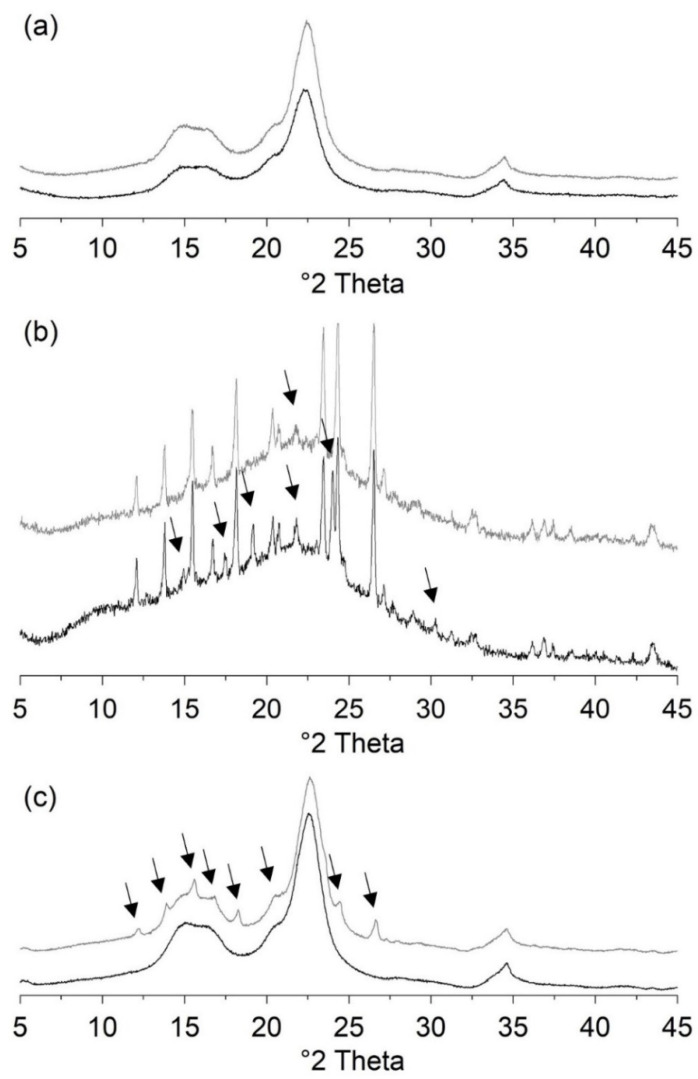
XRD studies of ASD tablets: (**a**) tablets of ASD VA30 with MCC 200 compressed at 20 kN and a dwell time of 1 s. Black curve: amorphous halo directly after preparation. Gray curve: amorphous halo after six months of storage at 40 °C/75% RH; (**b**) tablets of pure ASD VA40, compressed at 5 kN and a dwell time of 1 s. Black curve: diffraction peaks after six months of storage at 23 °C/75% RH. Gray curve: diffraction peaks after six months of storage at 40 °C/75% RH. The arrows indicate the diffraction peaks of PCM polymorphic form II; (**c**) tablets of ASD VA50 with MCC 200, compressed at 5 kN and a dwell time of 10 s. Black curve: amorphous halo directly after preparation. Gray curve: diffraction peaks of polymorphic form I after one week of storage at 23 °C/75% RH. The arrows indicate the diffraction peaks of PCM polymorphic form I.

**Figure 6:**
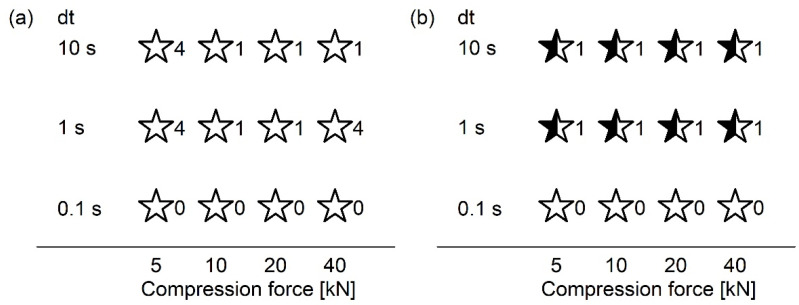
Stability of ASD tablets VA40 with MCC 200 during storage under two different conditions: (**a**) 23 °C/75% RH, (**b**) 40 °C/75% RH. Stars represent two types of recrystallization. Open stars show recrystallization in polymorphic form I. Semi-closed stars represent recrystallization in both form I and form II. The numbers indicate the weeks in which recrystallization was detected in the XRD measurements.

In XRD studies, tablets of ASD VA30 showed no diffraction peaks after six months of storage (Figure 5a). No differences were found in the presence or absence of MCC 200, under different storage conditions, compression forces, or with different dwell times. For tablets made of ASD VA40 with MCC 200 (Figure 6), which were produced at 0.1 s dt, small diffraction peaks were detected immediately after compression. Diffraction peaks in the diffractograms of three tablets stored at 23 °C (Figure 6a) were observed within one month. All of the other tablets displayed diffraction peaks within one week of storage. The tablets stored at higher temperatures showed more pronounced diffraction peaks of polymorphic form II (Figure 6b). Small diffraction peaks of polymorphic form II were also observed in tablets stored at 23 °C for compression forces of 20 and 40 kN after six months of storage.

The tablets without MCC 200 (Figure 5b), which were compressed at 5 kN, showed diffraction peaks immediately after production. For all other tablets, precise diffraction peaks could be found within one week of storage. The tablets stored at a relatively high temperature had almost only diffraction peaks of polymorphic form I after a storage period of three months. In comparison, the tablets stored at 23 °C still had polymorphic form II diffraction peaks after six months.

**Figure 7:**
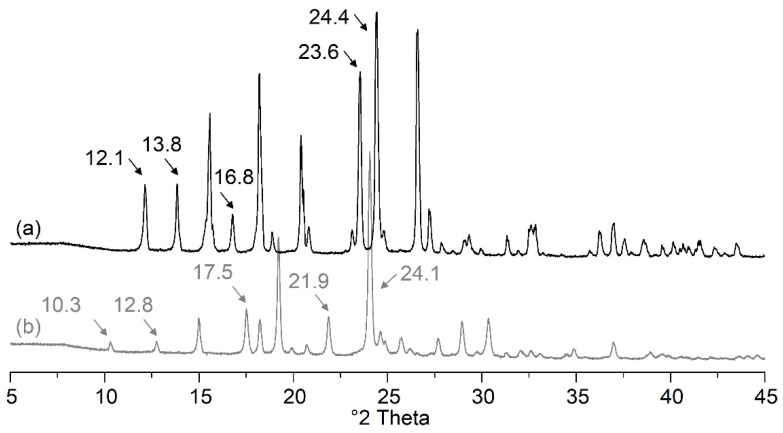
XRD diffractograms of PCM of different polymorphic forms. The arrows and numbers indicate the position of the characteristic diffraction peaks of each modification. (**a**) polymorphic form I, (**b**) polymorphic form II.

Tablets of ASD VA50 showed diffraction peaks in XRD studies within one week of storage under all conditions. The diffraction peaks could be assigned to polymorphic form I (Figure 5c). The XRD diffractograms of PCM of polymorphic forms I and II and their characteristic diffraction peaks are shown in Figure 7.

#### 3.2.3. Drug Release

**Figure 8:**
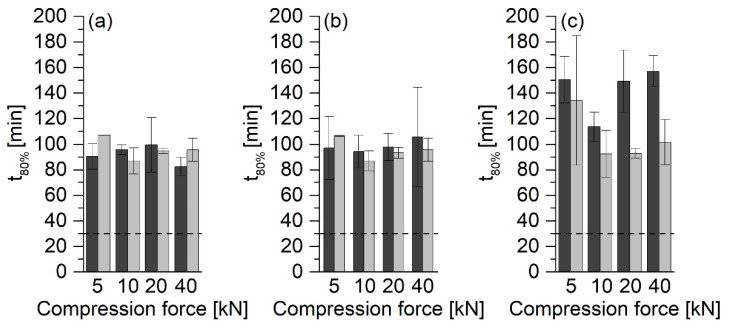
T_80%_ values of tablets without MCC 200. Dark gray bars: ASD directly after preparation; light gray bars: PM directly after preparation. (**a**) ASD/PM VA30 tablets, (**b**) ASD/PM VA40 tablets, (**c**) ASD/PM VA50 tablets, average ± SD, *n* = 2.

**Figure 9:**
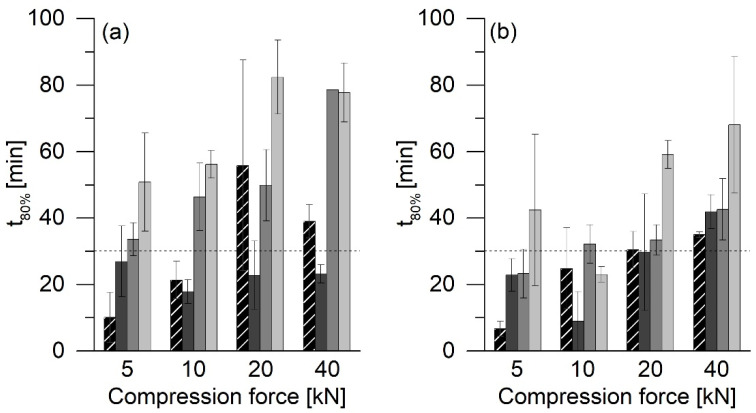
T_80%_ values of tablets with MCC 200 compressed at a dwell time of 1 s. Black-striped bars: ASD directly after preparation; dark gray bars: PM directly after preparation; gray bars: ASD after six months of storage at 23 °C/75% RH; light gray bars: ASD after six months of storage at 40 °C/75% RH. (**a**) ASD/PM VA30 tablets, (**b**) ASD/PM VA40 tablets, average ± SD, *n* = 2.

For a thorough comparison of the results, the t_80%_ values were determined for all of the tablet releases. In Figure 8 and Figure 9, the horizontal lines at 30 min indicate the USP requirement that PCM tablets must release at least 80% of the API within 30 min [14].

ASD tablets produced without filler showed high t_80%_ values. The t_80%_ values for tablets made of ASD VA30 (Figure 8a) and ASD VA40 (Figure 8b) ranged between approx. 80 and 100 min, regardless of the compression force. Tablets made of ASD VA50 had higher t_80%_ values that were between 110 and 160 min (Figure 8c). All PM tablets compressed at 5 kN and those prepared with ASD VA50 floated on the medium.

Compared to the ASD tablets without MCC 200, the freshly produced tablets with MCC showed significantly faster release, which depended on the compression force (Figure 9). The tablets that had been compressed at 5 kN had a t_80%_ value of fewer than 10 min, while the others showed much higher values that were between 25 and 40 min. The ASD tablets with VA30 compressed at 20 kN did not show this phenomenon. However, the large standard deviation must be taken into account.

Compared to the ASD tablets, the PM tablets that were compressed at 5 kN showed a larger t_80%_ value. For all of the other compression forces, this value was similar to or even lower than that for the ASD tablets. The tablets stored for more than six months showed a significant increase in the t_80%_ values compared to the freshly produced tablets. The tablets stored at 40 °C had the highest t_80%_ values at 50 to 80 min.

#### 3.2.4. SEM Images

To support the crushing strength results of the tablets, the ASD and PM tablets, and the tablets with and without MCC 200 were characterized by SEM.

**Figure 10:**
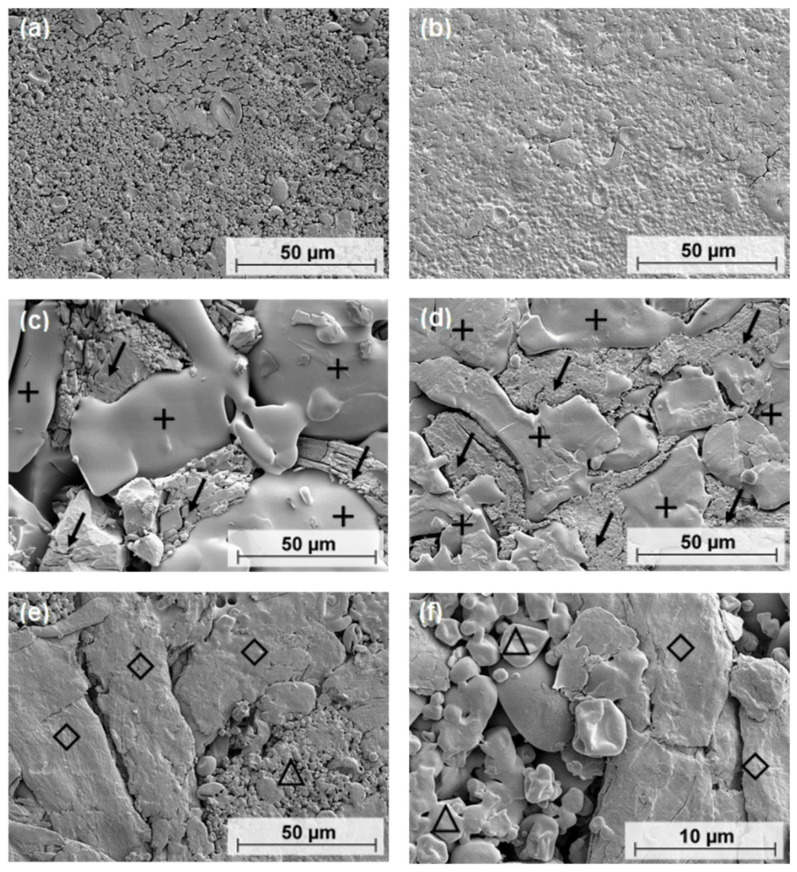
Scanning electron microscope (SEM) images of different tablets: (**a**) surface of pure ASD VA30 tablet, compressed at 5 kN; (**b**) surface of pure ASD VA30 tablet, compressed at 40 kN; (**c**) surface of pure PM VA30 tablet, compressed at 5 kN; arrows indicate PCM and crosses indicate PVP/VA; (**d**) surface of pure PM VA30 tablet, compressed at 40 kN; arrows indicate PCM and crosses indicate PVP/VA; (**e**,**f**) surface of ASD VA30 tablet with MCC 200, compressed at 10 kN; triangles indicate ASD VA30 and rhombuses indicate MCC 200.

The ASD tablets only showed one phase (Figure 10a,b) in comparison to the corresponding PM tablets, where two phases could be found (Figure 10c,d). ASD tablets compressed at 5 kN still showed some individual spherical particles, and a small gap remained between the particles (Figure 10a). Relatively high compression forces resulted in the intense deformation and fusion of the particles, making the surface more compact and denser (Figure 10b). In comparison, the PM tablets showed two distinctly separated phases. The structure consisted of a smooth phase (marked with a cross) and a phase with oblong particles (marked with an arrow) (Figure 10c,d). When a relatively high compression force was applied, the two phases remained separate, but the deformation of the oblong particles became stronger (Figure 10d).

ASD tablets with MCC 200 (Figure 10e,f) showed large, elongated particles of MCC (marked with a rhombus) and small, deformed spherical particles of ASD VA30 (marked with a triangle).

## 4. Discussion

### 4.1. Characterization and Stability of ASD Powders

PVP is known to stabilize the amorphous state of different APIs and has been the subject of several publications [15,16,17]. The ASD with PVP/VA tended to be the most unstable, with only minor stability differences observed when PVP 12, PVP 30, and PVP 90 were used. Independent of PVP length, ASDs containing up to 30% PCM remained amorphous for at least 48 weeks. However, the T_g_ differences between the products were up to 50 °C. Above a specific polymer concentration, saturation of the drug–polymer interaction can occur. Above this concentration, the stabilization of the API is no longer supported. The ASD prepared with PVP 12 tended to show the highest stability. Preparations with PVP/VA, on the other hand, showed the lowest stability. The recrystallization for the preparation with 50% PCM was predominantly in metastable polymorphic form II.

Pacult et al. found that with ASD from bicalutamide and three different PVP lengths, the preparations with the PVP with the longest chain showed the lowest stability. These authors justified this observation with the fact that the polymers have different free volumes between the polymer chains. This means that the polymer chains can assume different configurations in free space, which can either promote or reduce the stability of the samples. PVP 90, the polymer with the longest chain in this study, showed the highest free volume and the lowest stability [18]. Sekizaki et al. made similar observations regarding the recrystallization behavior of ASD from ibuprofen and PVP 90, PVP 30, and PVP 12 [19].

Mohapatra et al. observed opposite effects. In preparations with indomethacin, the stability of amorphous indomethacin could be improved by increasing the polymer chain length. These authors attributed this to the drug’s reduced molecular mobility due to the increase in PVP viscosity [20].

As PVP 30 and PVP/VA have a similar chain length, VA does not seem to be a more suitable recrystallization inhibitor than pure PVP.

It is assumed that PCM and PVP form a complex. As a mechanism of complex formation, van der Waals forces and hydrogen bridge bonds are discussed. Based on IR investigations, Garekani et al. showed that complex formation is probably based on a hydrogen bridge bond between the hydroxyl group of PCM and the carboxyl group of PVP [21]. Wen et al., on the other hand, said that the formation of hydrogen bridge bonds is sterically complicated. Instead, they assumed that the complex is formed by van der Waals forces [22].

Botha and Lötter [23] used DSC measurements to investigate ketoprofen compatibility with various excipients, e.g., PVP. They found that the absence of a melting peak for ketoprofen meant that interactions between the API and polymer could occur. We performed DSC measurements on the PM prepared with PCM and PVP/VA. The absence of the PCM melting peak was observed, which could also indicate an interaction between PCM and PVP/VA, e.g., complex formation.

The formation of hydrogen bonds can contribute to the stabilization of the amorphous state, as already reported for other drugs [24]. In comparison to pure PVP, PVP/VA also contains VA monomers. The bonds between VA compounds and the PCM may not be as strong, resulting in the polymer having less of a stabilizing effect. This may be a reason why the ASD prepared with PVP/VA shows the least stability.

VA could also be responsible for the fact that preparations with PVP/VA partially recrystallized in polymorphic form II. The occurrence of polymorphisms can be due to many different factors. VA may favor the recrystallization of PCM in polymorphic form II compared to pure PVP. Maniruzzaman et al. published similar results for preparations of PCM with Soluplus and PVP/VA. They achieved recrystallization of PCM in polymorphic form II at approximately 120 °C [25]. Rossi et al. studied preparations with hydroxypropyl methylcellulose (HPMC) and PCM. They also found that the crystallization of PCM in polymorphic form II or form III occurred through suitable thermal treatment [26].

A closer examination of the ASD prepared with PVP/VA and 40 to 50% PCM showed that up to 43% PCM, the samples recrystallized in polymorphic form I. For the ASD consisting of 44% PCM, diffraction peaks of polymorphic forms I and II were equally evident. In contrast, all other preparations predominantly showed diffraction peaks of form II. A relatively high PCM content for the given spray drying parameters led to recrystallization in polymorphic form II. These observations support the assumption that even small changes can vary the recrystallization tendency of an API. Nevertheless, further studies are still needed to fully understand the different recrystallization behaviors.

### 4.2. Characterization and Stability of ASD Tablets

As shown in Figure 3, all ASD tablets had higher crushing strengths than the corresponding PM tablets. PVP/VA has excellent properties as a dry binder in direct tableting. One of the reasons for this is because of the deformed structure of the hollow and spherical particles [27]. Particle bonding and compressibility are enhanced due to the large surface area [28]. The produced ASD powders also had an irregularly shaped structure, similar to pure PVP/VA. As a result, relatively strong interparticulate bonds can form between the particles, which can be one reason for the relatively high crushing strengths of the ASD tablets compared to PM tablets [29].

Furthermore, it has been reported that for lactose, for example, amorphous properties could improve compaction, which is likely due to the relatively strong plastic deformation of the amorphous particles and solid bridges [30,31].

The addition of 74% MCC 200 resulted in tablets with lower crushing strengths at lower compression forces than pure ASD tablets. MCC is known for its excellent plastic deformation and acts as a crucial dry binder. Nevertheless, MCC cannot achieve the properties of PVP/VA.

The stability of the ASD VA30 was not affected by tableting. The tablets, similar to the uncompressed powder, remained amorphous for at least 48 weeks. When evaluating the results, however, the detection limit for PCM in the XRD measurements must be considered. As described above, tablet formulations with MCC 200 only consist of 25% ASD. This means that tablets containing ASD with 30% PCM only consist of 7.5% PCM. We determined a PCM detection limit of approx. 1% for the XRD measurements. Partial recrystallization of PCM up to approx. 15% cannot be detected in this way and still appeared amorphous in the XRD measurements.

Some ASD VA40 tablets showed crystals of polymorphic PCM forms I and II, while the uncompressed powder only recrystallized in polymorphic form I. Therefore, the compression process might promote the recrystallization of the PCM in polymorphic form II. Smith et al. reported that they could achieve conversion to other polymorphic forms when the PCM of polymorphic form I was compressed at very high compression forces [11].

The ASD VA50 tablets with MCC showed recrystallization in polymorphic form I within one week. Initial analysis of the powders showed recrystallization in polymorphic form II. However, a later repetition of the powder stability also led to recrystallization in polymorphic form I within a week. Thus, tableting seems to have hardly any influence on the stability of ASD with PCM.

### 4.3. Drug Release of ASD Tablets

In the investigations, it was shown that the type of the ASD formulation has a significant influence on the release rate. The tablets with MCC 200 showed the fastest release. In contrast, the release rate of the tablets without MCC 200 was much slower, and hardly any differences between different compression forces could be found.

**Figure 11:**
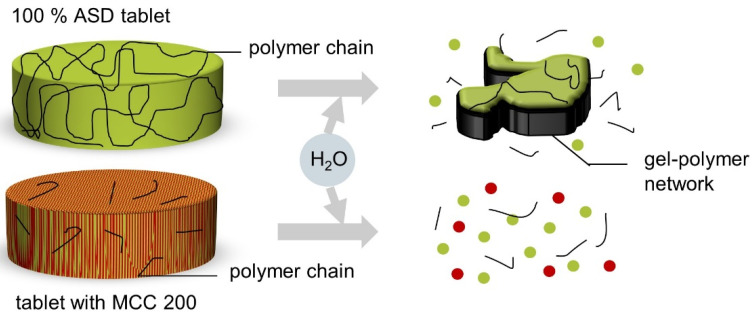
Illustration of the formation of a gel–polymer network in ASD tablets without MCC 200 and in comparison to tablets with MCC 200. Modified from Demuth et al. [5].

It has already been reported that tablets made of an ASD without other excipients show the slow release of the API [5]. This phenomenon is because the polymer chains form a gel–polymer network upon contact with water (Figure 11). Due to the longer path length through the gelatinous network, the tablet can only erode slowly, and a slow release of the API follows.

PVP/VA is used as a binder in tableting [27]. Increasing the binder content as well as increasing the compression force can lead to the increased formation of a gel network [5]. In this study, at compression forces of 5 kN, the particles are already compressed to such an extent that the compression force no longer appears to affect the release rate. Therefore, none of the formulations without MCC 200 met the USP requirement that 80% of the API must be released within 30 min. However, it must be considered that the tablets had similar crushing strengths. This observation applied to tablets produced with compression forces between 10 and 40 kN in particular.

ASD VA50 tablets showed even slower releases. The swelling of the PVP matrix in the release medium could trap air, which led to the buoyancy of the tablets. Thus, the medium could not thoroughly moisten the tablet surfaces, which led to a slower release of the API.

Compared to pure ASD and PM tablets, the MCC-containing tablets showed a faster release of the API. MCC is used as a filler and binder but also has the function of a disintegrant. The main mechanisms of MCC disintegration that are discussed are swelling [32] or wicking [33]. The high amount of added MCC prevented the formation of a gel network and supported the disintegration of the tablet so that the API was released more quickly. Nevertheless, faster PCM release from ASDs than from PMs could only be observed in tablets with MCC compressed at 5 kN.

After six months of storage under increased humidity and the subsequent drying of the tablets until a constant mass was achieved, post-curing was observed in almost all of the tablets (data not shown). The tablets made of ASD VA30 with MCC 200 showed a slower release of the API than tablets made of ASD VA40, although the ASD VA30 tablets remained amorphous within six months of storage. The higher PVP/VA content in the ASD VA30 tablets enhanced the formation of a gel–polymer network. Therefore, the release of the API from the ASD VA30 tablets was slower than that of the ASD VA40 tablets. Since the solubility of the amorphous PCM is only slightly higher than that of the crystalline form, the effect of the PVP/VA gel network is likely to be greater than the increase in the solubility of the amorphous PCM.

### 4.4. SEM

SEM could provide information on the morphology of ASD and PM tablets to support the crushing strength results. As the compressive force increased, the particles fused and lost their individuality. In contrast, the PM tablets showed two areas that were separated from each other. The smooth regions, where individual particles were not visible, were formed by PVP/VA. Crystalline PCM polymorphic form I is needle-shaped and forms an uneven phase. Thus, the homogeneous composition of ASD tablets may result in stronger interparticle bonds that are spread evenly throughout the tablet. In comparison, the PM tablets could only form strong bonds in parts of the tablet. The bonds between PVP/VA and PCM molecules may not be as strong as the bonds between ASD particles, resulting in the PM tablets having lower crushing strengths than the ASD tablets.

The tablets containing MCC 200 had lower crushing strengths than the tablets without an excipient. The average particle size of MCC 200 is 250 µm [34]. In contrast, ASD particle sizes are in the range of 5 to 10 µm. The PVP/VA in the PM tablets has a particle size of more than 50 µm in most cases. At low pressures, powders are less pre-compacted than they are at high pressures. Because of their different sizes, the particles in the tablets containing MCC 200 cannot approach each other in the same way as pure ASD particles. This results in a reduced number of binding sites and fewer interparticle bonds. This may explain the lower crushing strengths of the tablets made with MCC 200. In summary, tablets with smaller particles and smaller particle-size distributions tend to have higher crushing strengths. On the other hand, this tends to result in a slower release of the API.

## 5. Conclusions

The first part of the study showed that ASDs containing PCM and PVP 12, PVP 30, PVP 90, and PVP/VA could be produced using spray drying. Amorphous PCM could be stabilized up to a content of 30% for at least 48 weeks when formulated as ASD. Even under storage conditions of 40 °C and 75% RH, PCM remained amorphous, regardless of the polymer type used. Relatively high drug loadings resulted in recrystallization within a few days or weeks. Overall, ASDs with the copolymer PVP/VA showed the fastest recrystallization. On the other hand, differences in the PVP chain length had little effect on stability.

The compression of ASD powders can be problematic, as it can impair the stability of the amorphous state. In the second part of this study, it was shown that even the application of high compression forces and dwell times did not have a major impact on the stability of the amorphous state of PCM in ASDs. This suggests that tablets can be considered as a dosage form for these formulations. The ASDs also showed improved compression properties compared to the corresponding PM tablets. The ASD tablets had higher crushing strengths than the corresponding PM tablets. This allows for the compression force to be reduced during tableting.

## Figures and Tables

**Table 1:** Nomenclature of the different amorphous solid dispersion (ASD) powders. Example ASD powder 1230: The first two digits represent the polymer type (polyvinylpyrrolidone 12 (PVP 12), PVP/ vinyl acetate (PVP/VA)), and the last two digits represent the paracetamol (PCM) content (30%). The rest of the formulation consists of 70% polymer.

PCM Amount (%)	PVP 12	PVP 30	PVP 90	PVP/VA
10	1210	3010	9010	VA10
20	1220	3020	9020	VA20
30	1230	3030	9030	VA30
40	1240	3040	9040	VA40
50	1250	3050	9050	VA50

**Table 2:** Composition of tablets. Example: formulation K is physical mixture (PM) tablet. It consists of 10% paracetamol (PCM), 15% PVP/VA, 74% microcrystalline cellulose 200 (MCC 200), and 1% Mg-stearate.

Formulation	Ingredients (%)						
	ASD			PM			
	VA30	VA40	VA50	PCM	PVP/VA	MCC 200	Mg-Stearate
A	100	-	-	-	-	-	-
B	-	100	-	-	-	-	-
C	-	-	100	-	-	-	-
D	-	-	-	30	70	-	-
E	-	-	-	40	60	-	-
F	-	-	-	50	50	-	-
G	25	-	-	-	-	74	1
H	-	25	-	-	-	74	1
I	-	-	25	-	-	74	1
J	-	-	-	8	17	74	1
K	-	-	-	10	15	74	1
L	-	-	-	12.5	12.5	74	1

## Data Availability

The data are available upon request.
